# Positive Correlation Between Social Media Utilization by Orthopaedic Journals and Impact Factor

**DOI:** 10.5435/JAAOSGlobal-D-23-00009

**Published:** 2023-07-25

**Authors:** M. Kareem Shaath, Matthew S. Kerr, Jonathan D. Schwartzman, Frank R. Avilucea, Mark W. Munro, Joshua R. Langford, George J. Haidukewych

**Affiliations:** From the Orlando Health Jewett Orthopedic Institute, Orlando, FL (Dr. Shaath, Dr. Kerr, Dr. Avilucea, Dr. Munro, Dr. Langford, and Dr. Haidukewych); Florida State College of Medicine, Tallahassee, FL (Dr. Shaath, Dr. Avilucea, Dr. Munro, Dr. Langford, and Dr. Haidukewych); University of Central Florida College of Medicine, Orlando, FL (Dr. Shaath, Mr. Schwartzman, Dr. Avilucea, Dr. Munro, Dr. Langford, Dr. Haidukewych).

## Abstract

Social media use has exploded in popularity over the past decade with over 1.5 billion users on Facebook and 320 million users on Twitter. The aim of this study was to analyze the use of social media by orthopaedic journals and determine whether a relationship exists between social media followers and journal impact factor.

**Methods:**

The Clarivate Analytics Impact Factor tool was used to identify all orthopaedic journals with a 2022 impact factor of greater than 1.5. We then conducted a query on Instagram, Twitter, LinkedIn, and Facebook to determine which programs had pages on each platform.

**Results:**

Seventeen journals were included across all orthopaedic subspecialties. Of the 17 journals, 14 (82.4%) had a Facebook page, eight (47%) had an Instagram page, 15 (88.2%) had a Twitter account, and 8 (47%) had a LinkedIn profile. When compiling the number of followers by social media platform, Twitter had the most (177,543), followed by Facebook (149,388), Instagram (81,739), and LinkedIn (77,459). We found a significant correlation between the number of social media followers and journal impact factor (Pearson correlation coefficient [PCC] = 0.67; *P* = 0.003). When analyzing each social media platform independently, we found a significant correlation between the number of Facebook and Twitter followers and journal impact factor (PCC = 0.54; *P* = 0.02 and PCC = 0.80; *P* < 0.001, respectively).

**Discussion:**

We have shown a notable association between the number of social media followers and a journal's impact factor. With the increasing shift toward online distribution, orthopaedic journals may use our data when evaluating their social media strategy to maintain and potentially increase their exposure and potentially their impact factor.

Social media use has exploded in popularity over the past decade with over 1.5 billion users on Facebook and 320 million users on Twitter.^1^ Nearly 75% of all Americans use at least one social media platform, rising to over 90% of individuals between the ages of 18 and 29 years.^2^ The usage of social media by hospitals and physicians has also grown substantially.^3^ For the orthopaedic surgeon, social media has been found to assist with disseminating information, improving patient-physician connectivity, and practice building.^4,5^ Scholarly research has also seen a shift toward online distribution with discussions regarding published articles becoming increasingly digital and rapid after article publication.^6,7,8,9^ Online scholarly influence in orthopaedics has been found to occur largely on Twitter and Facebook.^1^

The aim of this study was to analyze the use of social media by orthopaedic journals and determine whether a relationship exists between social media followers and journal impact factor.

## Methods

The Clarivate Analytics Impact Factor tool was used to identify all orthopaedic journals with a 2022 impact factor of greater than 1.5. We then conducted a query on Instagram, Twitter, LinkedIn, and Facebook to determine which journals had pages on each platform. Once the social media accounts were identified, we used the first post to provide us with the start date for the account. In addition, we calculated the number of posts and noted the most recent post on the platform. This was repeated for each journal, independently. All social media websites were accessed on November 9, 2022. The data were then compiled into Microsoft Excel. Analyses were conducted using Minitab, and significance was set at *P* = 0.05.

## Results

Seventeen journals were included across all orthopaedic subspecialties (Table 1). Of the 17 journals, 14 (82.4%) had a Facebook Page (Table 2), eight (47%) had an Instagram page (Table 3), 15 (88.2%) had a Twitter account (Table 4), and 8 (47%) had a LinkedIn profile (Table 5). When compiling the number of followers by social media platform, Twitter had the most (177,543), followed by Facebook (149,388), Instagram (81,739), and LinkedIn (77,459).

**Table 1:** Included Journals and Associated Impact Factors

Journal Name	IF 2017-2018	IF 2018-2019	IF 2019-2020	IF 2021-2022	Mean IF
American Journal of Sports Medicine	6.1	6.1	5.8	7.0	6.2
Journal of Bone and Joint Surgery	3.6	4.3	4.6	5.4	4.5
Clinical Orthopaedics and Related Research	4.1	4.2	4.3	4.8	4.3
Arthroscopy	4.3	4.4	4.3	6.0	4.8
Journal of Arthroplasty	3.3	3.5	3.7	4.4	3.8
The Spine Journal	3.1	3.2	3.2	4.3	3.5
Journal of Shoulder and Elbow Surgery	2.8	2.9	2.8	3.5	3.0
Journal of Orthopaedic Research	3.4	3.0	2.7	3.1	3.1
Spine	2.8	2.9	3.2	3.2	3.0
Orthopedic Clinics of North America	2.7	2.5	2.4	2.8	2.6
Foot and Ankle International	2.7	2.3	2.3	3.6	2.7
Journal of the American Academy of Orthopaedic Surgeons	2.6	2.3	2.3	3.0	2.6
Journal of Hand Surgery	1.8	2.1	2.1	2.3	2.1
The Journal of Knee Surgery	2.1	1.6	2.0	2.5	2.0
Journal of Pediatric Orthopaedics	1.9	2.0	1.9	2.5	2.1
Journal of Orthopaedic Trauma	2.4	1.8	1.9	2.9	2.2
Clinical Spine Surgery	2.0	1.7	1.6	1.7	1.8

IF = impact factor

**Table 2:** Included Journals and Facebook Statistics

Journal Name	Date Created	Days Since Last Post	Total Followers	Total Page Likes
American Journal of Sports Medicine	May 2011	2	32,000	N/A
Journal of Bone and Joint Surgery	October 2011	2	28,953	26,794
Clinical Orthopaedics and Related Research	May 2011	2	9,400	8,900
Arthroscopy	September 2011	2	9,200	8,400
Journal of Arthroplasty	March 2011	5	2,300	2,100
The Spine Journal	N/A	N/A	N/A	N/A
Journal of Shoulder and Elbow Surgery	August 2011	0	6,239	5,792
Journal of Orthopaedic Research	June 2011	2	7,496	6,007
Spine	June 2011	1,788	2,100	2,000
Orthopedic Clinics of North America	N/A	N/A	N/A	N/A
Foot and Ankle International	December 2009	2	6,600	6,300
Journal of the American Academy of Orthopaedic Surgeons	April 2009	2	36,000	N/A
Journal of Hand Surgery	December 2015	5	1,100	874
The Journal of Knee Surgery	N/A	N/A	N/A	N/A
Journal of Pediatric Orthopaedics	October 2011	N/A	1,500	1,500
Journal of Orthopaedic Trauma	June 2011	1,828	2,200	2,100
Clinical Spine Surgery	October 2011	54	4,300	4,100
Totals			149,388	74,867

**Table 3:** Included Journals and Instagram Statistics

Journal Name	Date Created	Days Since Last Post	Number of Posts	Followers
American Journal of Sports Medicine	August 2020	2	403	5,366
Journal of Bone and Joint Surgery	March 2019	2	1742	23,700
Clinical Orthopaedics and Related Research	N/A	N/A	N/A	N/A
Arthroscopy	September 2019	2	1788	17,700
Journal of Arthroplasty	October 2020	5	365	6,126
The Spine Journal	N/A	N/A	N/A	N/A
Journal of Shoulder and Elbow Surgery	N/A	N/A	N/A	N/A
Journal of Orthopaedic Research	N/A	N/A	N/A	N/A
Spine	N/A	N/A	N/A	N/A
Orthopedic Clinics of North America	N/A	N/A	N/A	N/A
Foot and Ankle International	August 2019	2	796	4,973
Journal of the American Academy of Orthopaedic Surgeons	July 2014	7	712	20,900
Journal of Hand Surgery	December 2015	6	368	2974
The Journal of Knee Surgery	N/A	N/A	N/A	N/A
Journal of Pediatric Orthopaedics	N/A	N/A	N/A	N/A
Journal of Orthopaedic Trauma	N/A	N/A	N/A	N/A
Clinical Spine Surgery	N/A	N/A	N/A	N/A
Totals			6174	81,739

**Table 4:** Included Journals and Twitter Statistics

Journal Name	Date Created	Days Since Last Post	Number of Posts	Followers
American Journal of Sports Medicine	November 2012	2	1,820	41,400
Journal of Bone and Joint Surgery	March 2009	2	10,300	37,800
Clinical Orthopaedics and Related Research	May 2011	2	5,741	13,100
Arthroscopy	August 2012	4	4,032	19,600
Journal of Arthroplasty	June 2017	3	1,538	10,700
The Spine Journal	January 2010	2	4,559	10,600
Journal of Shoulder and Elbow Surgery	May 2012	1323	757	1,602
Journal of Orthopaedic Research	August 2021	2	492	948
Spine	N/A	N/A	N/A	N/A
Orthopedic Clinics of North America	September 2011	2	443	2,826
Foot and Ankle International	May 2019	2	847	2,200
Journal of the American Academy of Orthopaedic Surgeons	May 2010	2	516	19,700
Journal of Hand Surgery	June 2014	3	2,852	4,157
The Journal of Knee Surgery	N/A	N/A	N/A	N/A
Journal of Pediatric Orthopaedics	October 2011	2,252	2,814	151
Journal of Orthopaedic Trauma	June 2011	210	5,182	12,100
Clinical Spine Surgery	October 2011	551	3,079	659
Totals			44,972	177,543

**Table 5:** Included Journals and LinkedIn Statistics

Journal Name	Date Created	Days Since Last Post	Number of Posts	Followers
American Journal of Sports Medicine	N/A	N/A	N/A	N/A
Journal of Bone and Joint Surgery	N/A	7	N/A	23,751
Clinical Orthopaedics and Related Research	N/A	93	N/A	429
Arthroscopy	N/A	2	N/A	1,132
Journal of Arthroplasty	N/A	4	N/A	548
The Spine Journal	N/A	N/A	N/A	N/A
Journal of Shoulder and Elbow Surgery	N/A	N/A	N/A	N/A
Journal of Orthopaedic Research	N/A	N/A	N/A	N/A
Spine	N/A	N/A	N/A	N/A
Orthopedic Clinics of North America	N/A	N/A	N/A	N/A
Foot and Ankle International	N/A	2	N/A	10,081
Journal of the American Academy of Orthopaedic Surgeons	N/A	2	N/A	41,491
Journal of Hand Surgery	N/A	245	N/A	27
The Journal of Knee Surgery	N/A	N/A	N/A	N/A
Journal of Pediatric Orthopaedics	N/A	N/A	N/A	N/A
Journal of Orthopaedic Trauma	N/A	N/A	N/A	N/A
Clinical Spine Surgery	N/A	N/A	N/A	N/A
Totals				77,459

The date created and the number of posts are not available.

We found a significant correlation between the number of social media followers and journal impact factor (Pearson correlation coefficient (PCC) = 0.67; *P* = 0.003) (Table 6). We did not find a significant correlation between the number of posts and journal impact factor (PCC = 0.40; *P* = 0.13). When analyzing each social media platform independently, we found a significant correlation between the number of Facebook and Twitter followers and journal impact factor (PCC = 0.54; *P* = 0.02 and PCC = 0.80; *P* < 0.001, respectively). We did not find a significant correlation between the number of Instagram or LinkedIn followers and journal impact factor (PCC = 0.39; *P* = 0.12 and PCC = 0.23; *P* = 0.62, respectively).

**Table 6:** Journals' Overall Social Media Presence and Impact Factor

Journal Name	Total followers	Total posts	Mean IF
The American Journal of Sports Medicine	78,766	2,223	6.2
Journal of Bone and Joint Surgery	90,453	12,042	4.5
Clinical Orthopaedics and Related Research	22,500	5,741	4.3
Arthroscopy	46,500	5,820	4.8
Journal of Arthroplasty	19,126	1,903	3.8
The Spine Journal	10,600	4,559	3.5
Journal of Shoulder and Elbow Surgery	7,841	757	3.0
Journal of Orthopaedic Research	8,444	492	3.1
Spine	2,100	0	3.0
Orthopedic Clinics of North America	2,826	443	2.6
Foot and Ankle International	13,773	1,643	2.7
Journal of the American Academy of Orthopaedic Surgeons	76,600	1,228	2.6
Journal of Hand Surgery	8,231	3,220	2.1
The Journal of Knee Surgery	0	0	2.0
Journal of Pediatric Orthopaedics	1,651	2,814	2.1
Journal of Orthopaedic Trauma	14,300	5,182	2.2
Clinical Spine Surgery	4,959	3,079	1.8

IF = impact factor

## Discussion

The aim of this study was to determine whether there was a correlation between an orthopaedic journal's impact factor and their activity on social media. We did find a statistically significant correlation between impact factor and the number of social media followers.

A journal's impact factor is used to sort or rank it by relative importance.^10^ Journals with high impact factors publish articles that are cited more often than journals with lower impact factors.^11^ The journal impact factor is calculated by taking into account the average number of times articles from the journal are cited over 2 years.^11^ It is specifically calculated by dividing the number of citations by the total number of articles published in the previous 2 years.^11^ The greater exposure a journal has, the more likely their articles will be cited, increasing the journal's impact factor. Our results support this because there is a positive correlation between the number of followers on social media and a journal's impact factor. This is likely because of the increased exposure the journal receives because of a larger social media following.

A recently published study identified the top 100 social media influencers within orthopaedics and related their social media influence to academic influence.^5^ They found that social media influence was highly concordant with academic productivity as measured by the *h*-index.^5^ Our findings are similar as we found that a larger number of social media followers are associated with higher journal impact factors.

Evaniew et al^1^ published a study aimed to determine which types of online activities are most prevalent in orthopaedics, to identify potential factors associated with higher counts of online mentions and standard citations, and to explore a complementary approach to measuring overall scholarly influence on the basis of online activity and citations. They found that online scholarly influence in orthopaedics is dominated by activity on Twitter and Facebook and is associated with a longer time since publication, higher journal impact factor, less risk of bias, and higher author *h*-index values. In this study, the authors included randomized controlled trials and did assess individual journals independently. Regardless, our findings are similar because Facebook and Twitter are the main sites used by orthopaedic journals and an increased number of followers on those platforms are associated with a higher journal impact factor.

The main strength of our study is its novelty. To our knowledge, this is the first and only study in the literature to evaluate social media activity by medical journals. Furthermore, we are the first study to associate the number of followers on social media with an increased journal impact factor. The major weakness of our study is, although our findings are statistically significant, a journal's impact factor is likely a result of many factors, not just a following on social media.

In conclusion, we detail the use of social media by the top 17 orthopaedic journals across multiple social media platforms. In addition, we have demonstrated a statistically significant correlation between the overall number of social media followers and a journal's impact factor. Specifically, an increased number of followers on Facebook and Twitter are correlated with a higher impact factor. With the increasing shift toward online distribution, orthopaedic journals may use our data when evaluating their social media strategy to maintain and potentially increase their exposure and potentially their impact factor.
